# Histological and Biochemical Analysis after Posterior Mandibular Displacement in Rats

**DOI:** 10.3390/vetsci9110625

**Published:** 2022-11-10

**Authors:** Ioannis Lyros, Despoina Perrea, Konstantinos Tosios, Nikolaos Nikitakis, Ioannis A. Tsolakis, Efstratios Ferdianakis, Eleni Fora, Theodoros Lykogeorgos, Michael P. Maroulakos, Emmanouil Vardas, Maria Georgaki, Erofili Papadopoulou, Apostolos I. Tsolakis

**Affiliations:** 1 Department of Orthodontics, School of Dentistry, National and Kapodistrian University of Athens, 11527 Athens, Greece; 2 Laboratory of Experimental Surgery and Surgical Research “N.S. Christeas”, Medical School, National and Kapodistrian University of Athens, 11527 Athens, Greece; 3 Department of Oral Medicine & Pathology and Hospital Dentistry, School of Dentistry, National and Kapodistrian University of Athens, 11527 Athens, Greece; 4 Department of Orthodontics, School of Dentistry, Aristotle University of Thessaloniki, 54623 Thessaloniki, Greece; 5 “Hatzikosta” General Hospital of Messolonghi, 30200 Messolonghi, Greece; 6 Department of Orthodontics, Case Western Reserve University, Cleveland, OH 44106, USA

**Keywords:** mandibular posterior displacement, condylar growth, rat, class III malocclusion, orthodontic treatment, osteoprotegerin, RANKL, MCSF, condylar cartilage thickness

## Abstract

**Simple Summary:**

The lower jaw has a particular contribution to the human appearance, and so it is not uncommon for people to seek treatment in cases of its extreme growth. The orthodontist often selects to place intraoral devices in order to address the problem. This study used healthy young rats to guide their lower jaws backward. Measurements of biochemical molecules in blood that are related to bone metabolism did not reveal any statistically significant differences. However, noteworthy local microscopic alterations of the condyle were evidenced. They involved histological changes in bone structure and the thickness of cartilage. In conclusion, it seems that the procedure under study might not cause any severe organic disturbance in the lab animals under the conditions of the experiment. Only minor histological effects on condylar morphology were observed.

**Abstract:**

The present study aimed to investigate any biochemical and histological changes of the rat condyle and mandible in animals that had sustained mandibular growth restriction. Seventy-two male Wistar rats were divided into two equal groups, experimental and control. Each group consisted of three equal subgroups. The animals were sacrificed 30, 60, and 90 days after the start of the experiment. Blood samples were collected from the eye, and the osteoprotegerin (OPG), Receptor Activator of Nuclear Factor Kappa B Ligand (RANKL), and Macrophage Colony-Stimulating factor (MCSF)concentrations were measured by using enzyme-linked immunosorbent assay (ELISA) kits. A histological analysis was performed on the mandibular condyles. The blood serum values of OPG, RANKL, and MCSF did not exhibit any statistically significant difference between groups or subgroups. However, significant histological changes became evident after a histomorphometric condylar examination was performed. The Bone Surface/Total Surface ratio appeared reduced in the anterior and posterior regions of the condyle. In addition, the Posterior Condylar Cartilage Thickness was measured and determined to be significantly diminished. The present intervention that employed orthodontic/orthopedic devices did not prove to have any significant effect on the circulating proteins under study. Posterior displacement of the mandible may culminate only in local histological alterations in condylar cartilage thickness and its osseous microarchitecture.

## 1. Introduction

In humans, the mandible is connected to the skull by the two temporomandibular joints (TMJ) and moves in relation to the skeletal base, as guided by muscular attachments contracting after voluntary or involuntary neural stimulation [1]. The mandibular condyle performs translatory and rotary movements that are cushioned by a fibrocartilaginous articular disc with disparate thicknesses [2]. In the TMJ, the articular surfaces are load bearing and are lined with dense, avascular, fibrous connective tissue [3].

The fibrocartilaginous complex and the underlying trabecular bone constitute a functional entity withstanding the mechanical tension produced by mandibular function [4]. Bone continuously remodels to provide structural support to the articular structures during jaw movement [5,6]. The localized architecture and density supposedly accommodate the load on the cartilage, and so they partially reflect the metabolic condition of the TMJ [7].

The cartilage on the condylar head features four distinct layers, namely the fibrous, proliferative, mature, and hypertrophic, as identified from the articular surface to the underlying bony foundation. The fibrous zone contains fibroblast-like flat cells, the proliferative zone has heterogeneously distributed mesenchymal precursor cells, and the differentiated chondrocytes may be traced in the mature and hypertrophic zones [3,7].

Bone resorption is an essential component of the development and remodeling of the skeletal substrate [8]. In the abovementioned process, the predominant cell appears to be the osteoclast [9]. The mononucleated precursors invade the mesenchyme surrounding rudimentary bone to proliferate, differentiate into tartrate-resistant acid phosphatase (TRAP)-positive cells, and migrate in conjunction with endothelial cells [10,11,12]. Subsequently, they invade the cartilage to transform into mature multinucleated osteoclasts [9,13]. These specialized resorptive cells evolve from hematopoietic stem cells under the control of various systemic and local factors [14,15]. Osteoclast differentiation factor [12,16], RANKL [17], OPG [18], M-CSF [19], Vascular Endothelial Growth Factor (VEGF) [10,20], Interleukin-1 (Il-1) [21], and tumor necrosis factor (TNF-α) [22] are all implicated in osteoclastic function and the metabolism of the osseous tissue. Moreover, proteinases of the matrix metalloproteinase (MMP) family appear to exert direct chemotactic activity on osteoclasts for their recruitment [23].

The present study investigated the serum levels of RANKL, MCSF, and OPG proteins, as well as histomorphometrical changes effected in the condylar cartilage and trabecular bone, following distal functional displacement of the mandible in the rat. Those findings may be utilized in explaining systemic and regional disturbances of the animals that can subsequently be extrapolated into clinical practice in human patients.

## 2. Materials and Methods

The protocol of the experiment received approval by the Veterinary Directorate under the number 598742/04-10-2019 and was registered as EL 25 BIO 05, in agreement with Greek national legislation (P.D 56/2013) and in line with the European Directive 2010/63/EE and the European Council (276/33/20.10.2010), which refer to vertebrate experimental animal protection. The protocol is related to a multifaceted study, and the animal sample is common [24].

### 2.1. Experimental Design

Seventy-two (72) four-week-old male Wistar rats were studied. They were all bred for four weeks at the Hellenic Pasteur Institute before subsequently being transferred and housed at the Laboratory for Experimental Surgery and Surgical Research “N. S. Christeas” at the University Laboratory, School of Medicine, Athens, Greece. Cages (Tecniplast S.P.A., Buguggiate, Italy) were selected in accordance with National and European legislation. They were centrally ventilated at 15 air changes per hour in a stable environment (55% relative humidity; temperature at 20 ± 2 °C) and artificial alternating 12-hour cycles of light and dark.

The allocation of the animals into equal groups, experimental (Group A) and control (Group B), was achieved in random, using a digital tool (Random Team Generator). Each group was further divided into three subgroups including twelve animals each (A1, A2, A3, B1, B2, and B3).

As previously described [25], partially modified intraoral orthodontic appliances were cemented to the upper experimental rat incisors to promote backward displacement of the mandible. They were full-cast metallic and were produced in the lab. Their fabrication was based on a previous digital intraoral scanning (TRIOS 3, 3Shape intraoral scanner) of a randomly picked rodent. Zinc phosphate cement (Harvard Cement Normal Setting; Harvard Dental International GmbH, 15366 Hoppegarten, Germany) was selected for cementation. Throughout the period of study, animals were provided ad libitum with water and mashed food, a blend of pellets with water in a well-defined ratio, achieving a porridge-like texture.

Altogether, the duration of the experiment was 90 days. Animals were euthanized after 30 (Subgroups A1 and B1), 60 (Subgroups A2 and B2), and 90 (Subgroups A3 and B3) days. At the 60th experimental day, appliances were debonded from Experimental Subgroup A3. During the experiment, rats remained under close monitoring to ensure optimal development and growth.

### 2.2. Biochemical Analysis

**Figure 1:**
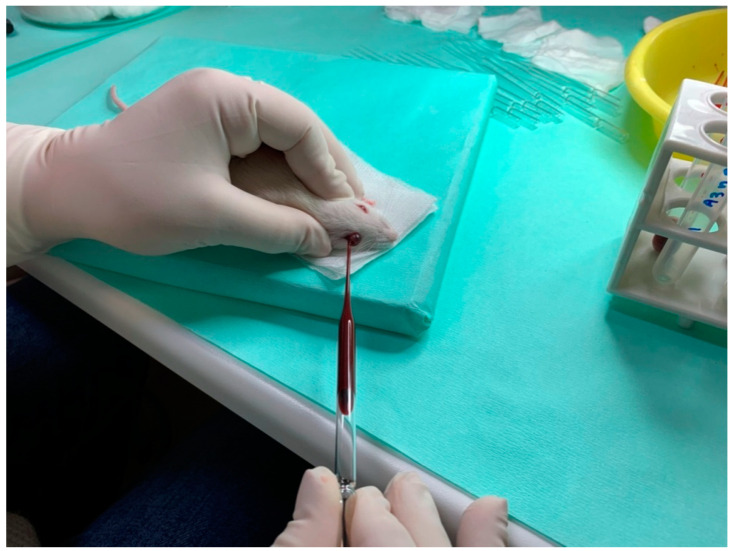
Blood collection.

To measure the circulating levels of RANKL, MCSF, and OPG proteins, rat serum was collected. On day 1 (initial) and the day of sacrifice (final) of the experiment, blood collection was performed. The animals were brought into general anesthesia by intramuscular injection of a ketamine–xylazine solution (0.2 mL/kg). Moreover, an ether chamber was used occasionally, strictly limited to terminal blood collection. Blood samples were collected from the eye with a thin sterile laboratory pipette, which was inserted behind the eyeball. Blood was transferred in tubes containing heparin and centrifuged at 13,000 rpm for 5 min at room temperature. Subsequently, the serum was collected and stored at −20 °C to be further analyzed. Proteins were evaluated using enzyme-linked immunosorbent assay (ELISA) kits (Elabscience^®^, Houston, TX, USA), following the manufacturer’s guidelines (Figure 1).

These ELISA kits use the Sandwich-ELISA principle. In brief, 100 μL of serum and the protein standards were added to precoated micro-ELISA plates containing the corresponding antibody specific to each protein. Then a biotinylated detection antibody specific for each protein and Avidin Horseradish Peroxidase (HRP) conjugate were added successively to each micro-plate well and incubated. When substrate solution was added to the wells, the wells containing each protein turned blue and the intensity of blue color was proportional to the amount of protein contained in the sample. Once the reaction was stopped with a stop solution, the color changed to yellow, and the optical density (OD) of yellow color was estimated spectrophotometrically at 450 nm. The concentration of the protein in each sample was calculated by comparing the OD values of the samples to the standard curve, which was created by using OD values of the known concentration of the standards for each protein.

The measurements were carried out in an ELISA photometer (Thermo Scientific Multiskan GO Microplate Spectrophotometer, Waltham, MA, USA). The ELISA tests were performed following the manufacturer’s instructions.

### 2.3. Histomorphometry

Before recovery, the animals were sacrificed by cervical dislocation. Next, their heads were dissected, and the soft tissues were removed carefully. Each head, intact and separately, was fixed in 10% formalin solution. 

**Figure 2:**
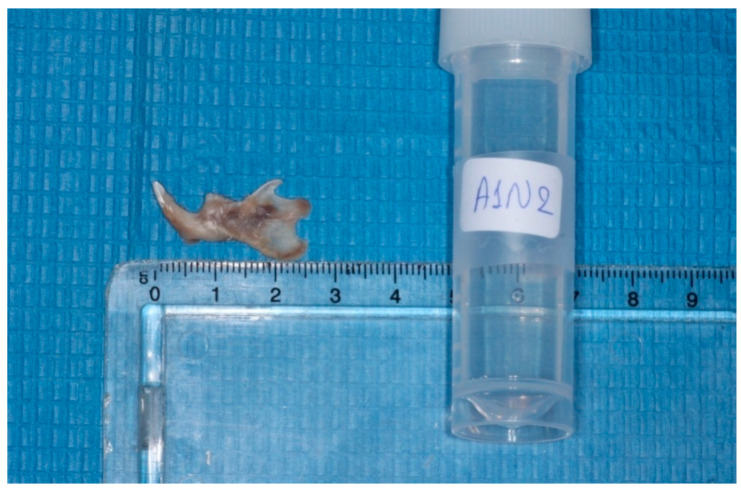
Isolation of the mandibles.

**Figure 3:**
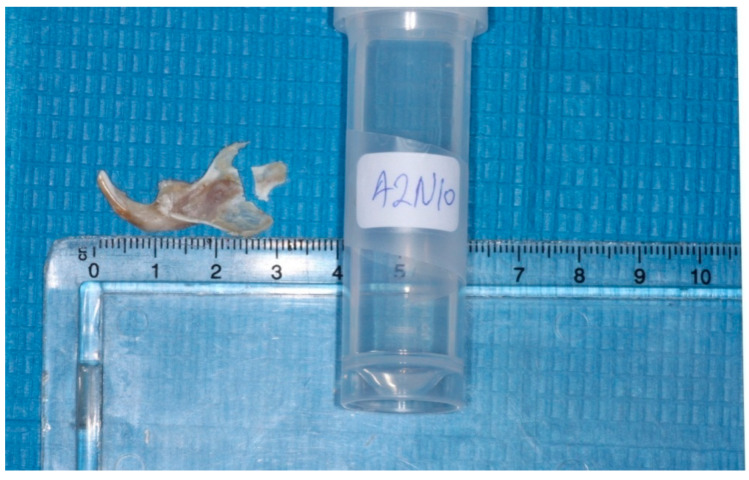
Isolation of the condyles.

The heads were cut in the middle, the left mandibles were separated from the heads, and the condyles were isolated (Figure 2 and Figure 3). Each condyle was decalcified in ethylene diamine tetra-acetic acid (MICRODEC EDTA-BASED, DIAPATH S.p.A, Martinengo BG, Italy) solution for 10 days and embedded in paraffin, using conventional methods. Then 6 μm–thick serial sections were cut by using a fully motorized rotary microtome (ARM3600, Histo-Line Laboratories Co., Pantigliate, MI, Italy) parallel to the sagittal plane of the mandibular condyle. The sections were stained with hematoxylin and eosin (H&E) to observe potential histomorphological changes.

The H&E sections were scanned at ×20 magnification with an Olympus CX23 RFS2 microscope (Olympus, Tokyo, Japan) equipped with a digital camera (Leica DC300F, Leica Microsystems AG, Heerbrugg, Switzerland). The TIFF image files generated were then converted to JPEG for the purposes of digital analysis, using the software Sedeen Viewer Version 5.4.4. (Copyright © Pathcore Inc., 2008–2019, Toronto, ON, Canada).

**Figure 4:**
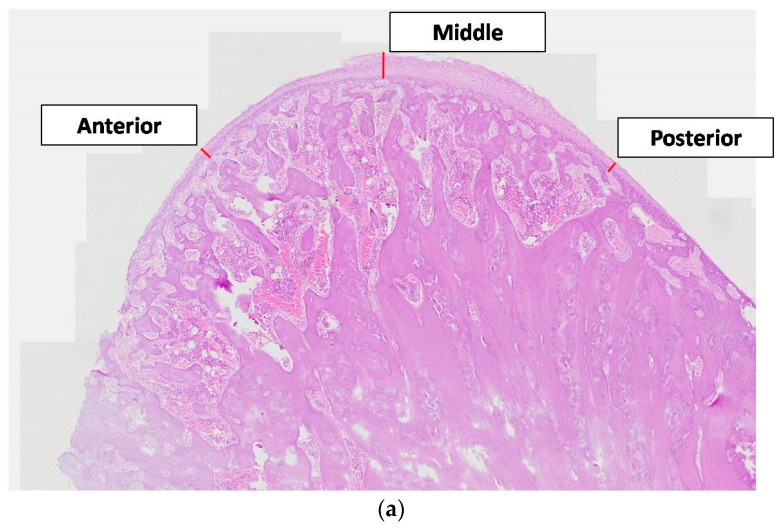

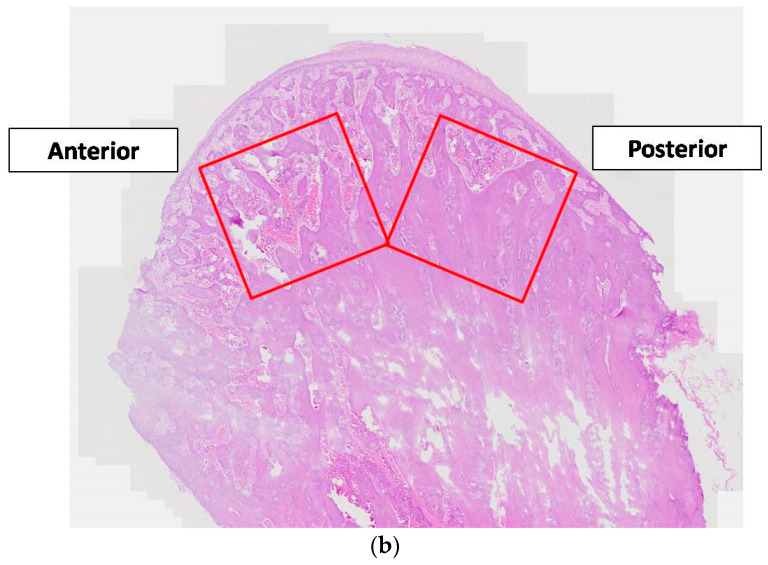
Histological images of the sagittal sections of the condyle (**a**). Condylar cartilage thickness measurements (anterior, middle, and posterior). (**b**). Square condylar head regions of interest (anterior and posterior), measuring 1.0 × 1.0 mm (H&E staining, original magnification ×20).

The thickness of the articular cartilage was considered at three areas, at both ends (anterior and posterior), and the midline across the sagittal plane passing through the condylar head. All cartilaginous layers (fibrous, proliferating, mature, and hypertrophic) in the anterior, middle, and posterior areas were identified and measured with a linear calculation tool (Image-Pro Plus v6.0.0.260 Media Cybernetics, Inc., Rockville, MD, USA©), from the lowest border of the hypertrophic layer up to the outer border of the fibrous layer (Figure 4a).

To calculate the Bone Surface/Total Surface ratio, two square condylar head regions of interest (anterior and posterior), measuring 1.0 × 1.0 mm, were selected for image analysis (Figure 4b). The ratio was measured with a digital tool (Image-Pro Plus v6.0.0.260 Media Cybernetics, Inc., Rockville, MD, USA©).

### 2.4. Statistics

Each animal group size was determined after performing a power analysis to be kept sufficiently small, for ethical reasons, but adequate to reliably detect any potential statistically significant results. Moreover, the size of the studied subgroups was finalized after considering the low probability that some rodents could perish due to a potentially stressful experimental manipulation. Standard statistical criteria (a = 0.05; b = 0.10), yielding a power of 90%, led to the allocation of 12 rats in each subgroup. Thus, 72 rats in total were recruited, allocated into the aforementioned Groups A and B.

One-way ANOVA or Kruskal–Wallis testing was performed for comparisons by timing. Differences in markers by group and timing were investigated by using regression models with each marker’s change from the initial measurement (final–initial) as the dependent variable and group and using timing and their relation as independent ones. Estimates were adjusted for multiple comparisons, using the Bonferroni method.

An analysis was performed at α = 5% level of statistical significance (*p*-value < 0.05). Statistical software Stata ver.14 (Stata Statistical Software: Release 14. StataCorp LP., College Station, TX, USA) was used for the coding and analysis of data.

## 3. Results

### 3.1. Biochemical Findings

**Figure 5:**
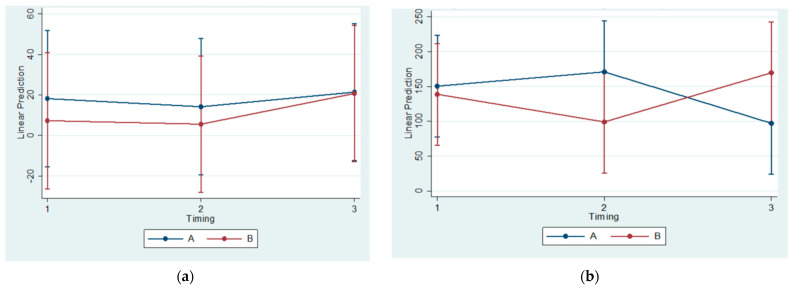

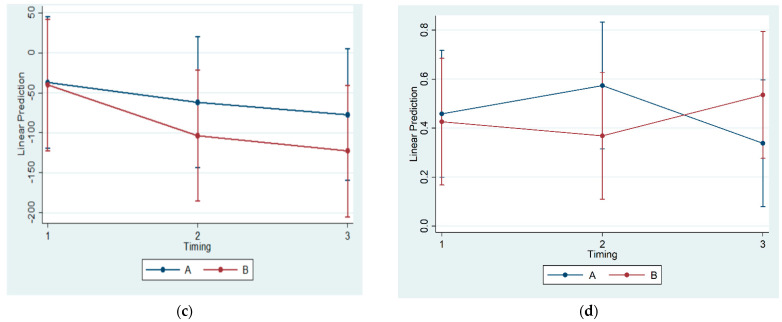
Estimated mean change (final–initial) and 95% confidence interval per group and timing in RANKL (**a**), OPG (**b**), MCSF (**c**), and OPG–RANKL ratio (**d**).

Tables with descriptive statistics for each serum marker and graphs with estimated means changes (final–initial value) and 95% confidence intervals are provided (Table 1, Table 2, Table 3 and Table 4 and Figure 5).

No statistically significant difference was observed regarding groups or timing. However, statistically significant increases were observed in the OPG and OPG/RANKL ratio within each subgroup, considering final and initial values. Contrarily, a statistically significant reduction was found in the MCSF in Subgroups B2 and B3.

### 3.2. Histomorphometric Observations

**Figure 6:**
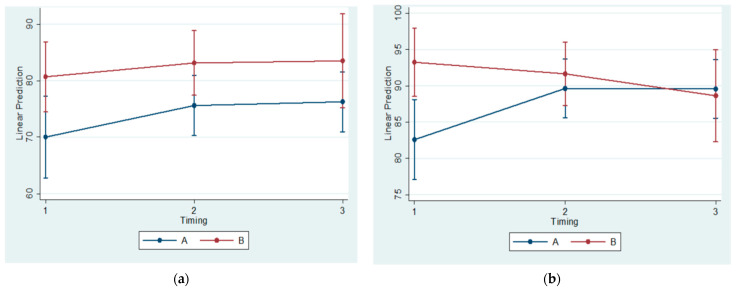

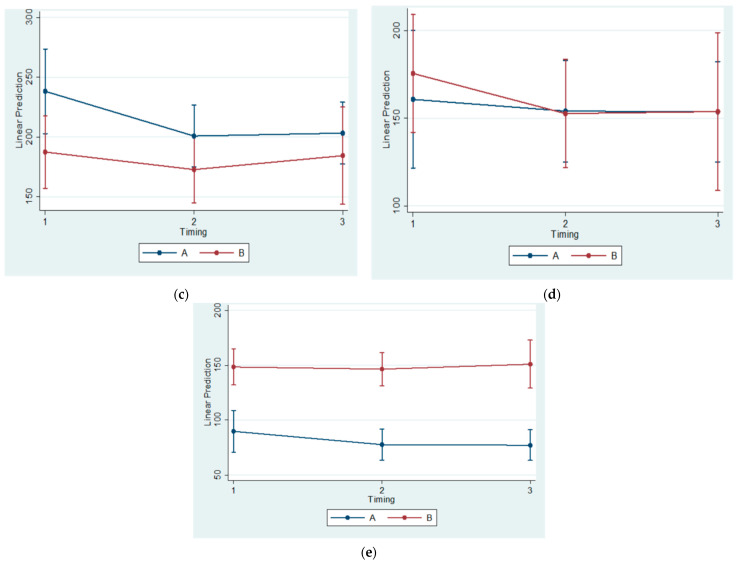
Estimated mean and 95% confidence interval per group and timing in Anterior Ratio Bone Surface/Total Surface (**a**), Posterior Ratio Bone Surface/Total Surface (**b**), Anterior Condylar Cartilage Thickness (**c**), Middle Condylar Cartilage Thickness (**d**), and Posterior Condylar Cartilage Thickness (**e**).

Descriptive statistics and estimated means (with 95% confidence intervals) are provided in Table 5, Table 6 and Table 7 and Figure 6.

The ratio of the Bone Surface/Total Surface (BS/TS) was found to be statistically significantly lower at both the anterior and posterior areas between Subgroups A1 and B1. In these subgroups, the Anterior Condylar Cartilage Thickness (Anterior CCT) was measured to be statistically significantly greater.

In addition, statistically significantly lower values were evidenced between experimental and control groups in regard to the Posterior Condylar Cartilage Thickness (Posterior CCT) at 30, 60, and 90 days.

## 4. Discussion

The orthodontist aims to correct malocclusion and to guide facial proportions into harmony [26]. Thus, the treatment proceeds to alter an established balance, albeit undesirable, and this might annoy the individual topically or even provoke a more pervasive discomfort, as evidenced in complications reported in various other minor and major aesthetic interventions [27,28,29].

Mandibular displacement is related to bone remodeling [24,30,31]. One of the aims of this study was to uncover any alteration in circulating biomolecules that are connected to osseous turnover which might reveal the dissemination of unwanted effects throughout the organism with ensuing dysregulation.

For this purpose, an experiment was conceived and conducted with the aid of lab animals, namely Wistar rats. The rat has been established as a popular model for the study of developmental issues [32], despite existing anatomical differences that might generate problems when extrapolating the conclusions into humans and checking their validity [33].

Experimental procedures (appliance bonding, blood sampling, and animal sacrifice) were achieved while the rats were being kept under general anesthesia. The state of unconsciousness was selected with the aim to decrease the anticipated animal agitation. The main anesthetic agent was ketamine–xylazine in combination. Ether was also used occasionally, notwithstanding its potentially severe side effects. However, the practice remained strictly confined in terminal blood sampling, so as not to affect target biomolecule values and animal welfare [34,35]. Throughout the experiment and postoperatively, all animals were kept under close monitoring for health issues and signs of discomfort. The rats gained weight as expected.

As every possible effort was made to acquire a homogenous sample, the rats were all male and of the same age, as has been done by others [36,37]. The gender of the experimental animals could have been a confounding factor, as the same animals were scheduled for research on miscellaneous issues related to growth [24]. Therefore, further research on the molecules of interest involving female animal models may be warranted. The living conditions of the animals were calibrated, and also their breeding remained strictly standardized, as the diet in particular might differentially alter mandibular growth and cause systemic errors, as has been shown by Tsolakis et al. (2019), Karamani et al. (2022), and Tsolakis et al. (2022) [38,39,40].

No statistically significant correlations of OPG, RANKL, and MCSF concentrations in blood serum were found across all subgroups. Thus, a systemic effect of posterior mandibular functional displacement cannot be substantiated by the results of the present study. Some authors have claimed that posterior mandibular displacement causes inflammation. Cholasueksa (2004) found that the procedure was injurious, as evidenced by the re-emergence of immunoreactive nerve fibers [36]. Similarly, Figueroba et al. (2014) [41] reported increased levels of pro-inflammatory molecules in the synovial tissues following functional stress application on the TMJ of rats. However, both studies referred only to region-specific effects.

The histomorphometrical observations of the present study agree with the results of previous ones. The statistically significant differences between Subgroups A1 and B1(referring to the 30th day of the experiment) regarding the BS/TS Ratio confirm the findings of Kuroda et al. (2011) [4], who recruited sixteen male Wistar rats of similar age (5-week-old compared to 4-week-old animals in our study) that were also divided into experimental (eight rats having intraoral functional appliances cemented with composite resin on their maxillary incisors) and control groups (eight rats seemingly without appliances). In the above study, the appliances were manufactured from orthodontic band material, whereas in the current study, the appliances were fully metallic based on a digital impression. The animals were fed powered food, which seems comparable to the diet consistency in the current study (porridge-like). However, their experiment lasted for 14 days compared to 90 days of the present study. On the other hand, they employed microcomputed tomography (micro-CT), along with histological techniques, to investigate cancellous bone three-dimensional structural changes.

Moreover, the reduction of the condylar cartilage width at the posterior region was identified by Teramoto et al. (2003) [42], Cholasueksa et al. (2004) [36], and Hua et al. (2012) [37]. Among them, Teramoto et al. (2003) [42] used somewhat older (8-week-old) male Wistar rats. The functional force to provoke mandibular retrusion was applied on mandibular incisors via a neck collar connected to bilateral jigs with properly designed springs. The animals’ food (hard) differed from what we provided the animals with, and this might have had repercussions on the mandibular dimensions, as was mentioned by Karamani et al. (2022) and Tsolakis et al. (2022) [39,40]. Teramoto et al. (2003) divided the rats into experimental (17 animals) and control (7 animals not been treated). [42] The animals were followed for 7 days at most (experimental and control groups). Cholasueksa et al. (2004) also selected male Wistar rats of 8 weeks of age, in line with Teramoto et al., as outlined previously [36]. Their sample included 39 rats, which were randomly allocated into experimental and control groups. The functional posterior mandibular displacement was affected by an intraoral guiding device cemented on the maxillary incisors with composite resin for a period lasting up to 14 days. The animals were provided with standard food and water ad libitum. Hua et al. (2012) experimented with 48 6-week-old male Wistar rats that were allocated into experimental and control groups [37]. The observation lasted for 3, 14, 30, and 60 days, depending on the respective subgroup. The food consisted of normal rat pellets and water. Backward mandibular movement was eventually realized with a custom-made, intraoral, full metallic twin inclined plane device that was properly cemented on maxillary and mandibular posterior teeth to cause distal displacement of the lower jaw.

Contrarily, Figueroba et al. (2014) observed thickening of the four cartilage layers in the middle region of the experimental condyles that were undergoing functional posterior displacement, in comparison to controls, after 14 days of intervention [41]. The present study, in agreement with previously published research [4,36,37,42], identified a reduction of the condylar cartilage thickness at the posterior region. Consequently, it might be concluded that the horizontal component of the chewing force is transmitted to the posterior condylar region.

Nevertheless, Ingervall et al. (1972) used 47 male Sprague-Dawley rats divided into three subgroups according to their age at the beginning of experiment (5, 13, and 52 weeks of age). The mandibles were displaced backward by an inclined metallic plane cemented on the lower incisors for 14 days. They alleged that the experimental mandibular retrusion promoted marked tissue reactions in the TMJ, which enhanced increased formation of cartilage in the posterior area of the condyle [43].

This study was conducted with rats, the most popular experimental animal model [30,44]. A long-term follow-up of the animals was fundamental to investigate the stability of results. Further research could evaluate the local status of markers related to bone metabolism by utilizing immunohistochemical techniques.

## 5. Conclusions

In the present study, functional posterior mandibular displacement in rats by a local procedure did not alter the serum levels of RANKL, MCSF, and OPG proteins involved in bone metabolism. Seemingly, this procedure has the potential only to cause localized changes in the cartilage thickness and the osseous microstructure of the condyle.

## Figures and Tables

**Table 1:** Descriptive statistics (mean and standard deviation) for each measurement by subgroups and overall for the experimental (Group A) and control (Group B) groups.

Descriptive Statistics	Mean (SD)				
Experimental Group A	Subgroups—Timing				
	**A1—0 d**	**A2—0 d**	**A3—0 d**	**Overall**	***p*** **-Value**
RANKL (pg/mL)	275.45 (49.00)	289.02 (47.62)	285.12 (51.62)	283.20 (48.36)	0.788
OPG (pg/mL)	110.37 (46.96)	133.67 (65.64)	117.05 (27.81)	120.36 (48.88)	0.498
MCSF (pg/mL)	314.45 (173.31)	229.19 (111.97)	226.27 (103.80)	256.64 (135.97)	0.200
OPG/RANKL ratio	0.40 (0.17)	0.45 (0.17)	0.42 (0.13)	0.42 (0.15)	0.775
	**A1—30 d**	**A2—60 d**	**A3—90 d**	**Overall**	***p*** **-Value**
RANKL (pg/mL)	293.64 (64.05)	303.22 (38.40)	306.50 (60.05)	301.12 (54.01)	0.840
OPG (pg/mL)	260.84 (183.64)	304.52 (121.77)	214.12 (123.69)	259.83 (146.52)	0.328
MCSF (pg/mL)	277.31 (126.81)	167.28 (69.35)	148.72 (83.99)	197.77 (109.98)	0.005 *
OPG/RANKL ratio	0.86 (0.57)	1.02 (0.42)	0.76 (0.51)	0.88 (0.50)	0.445
**Control Group B**	**Subgroups—Timing**				
	**B1—0 d**	**B2—0 d**	**B3—0 d**	**Overall**	***p*** **-Value**
RANKL (pg/mL)	298.60 (38.86)	279.33 (25.34)	284.08 (43.13)	287.34 (36.47)	0.415
OPG (pg/mL)	121.94 (60.25)	97.29 (50.91)	112.28 (56.86)	110.50 (55.48)	0.561
MCSF (pg/mL)	335.30 (188.65)	276.49 (169.91)	279.70 (145.37)	297.16 (166.28)	0.635
OPG/RANKL ratio	0.41 (0.21)	0.35 (0.17)	0.39 (0.18)	0.38 (0.18)	0.711
	**B1—30 d**	**B2—60 d**	**B3—90 d**	**Overall**	***p*** **-Value**
RANKL (pg/mL)	305.90 (56.14)	285.00 (47.53)	304.79 (64.11)	298.56 (55.56)	0.597
OPG (pg/mL)	260.60 (120.48)	196.22 (69.83)	281.85 (139.57)	246.22 (116.53)	0.175
MCSF (pg/mL)	294.98 (176.11)	172.66 (134.08)	156.83 (69.58)	208.15 (144.36)	0.032 *
OPG/RANKL ratio	0.83 (0.28)	0.72 (0.29)	0.93 (0.43)	0.83 (0.34)	0.327

* A1 vs. A2 *p* = 0.026, A1 vs. A3 *p* = 0.008, * B1 vs. B3 *p* = 0.050 (Bonferroni corrected).

**Table 2:** Estimated mean changes (final–initial), 95% confidence intervals and *p*-values (comparing with 0, i.e., no change) per group and timing.

Final–Initial	Mean Change	95% Confidence Interval	*p*-Value
**RANKL (pg/mL)**			
A1	18.19	−15.47, 51.86	0.285
B1	7.30	−26.36, 40.97	0.666
A2	14.20	−19.47, 47.87	0.403
B2	5.67	−28.00, 39.34	0.738
A3	21.38	−12.29, 55.04	0.209
B3	20.71	−12.96, 54.38	0.224
**OPG (pg/mL)**			
A1	150.48	77.45, 223.51	**<0.001**
B1	138.66	65.63, 211.69	**<0.001**
A2	170.86	97.83, 243.89	**<0.001**
B2	98.94	25.91, 171.97	**0.009**
A3	97.07	24.04, 170.10	**0.010**
B3	169.57	96.54, 242.60	**<0.001**
**MCSF (pg/mL)**			
A1	−37.15	−119.23, 44.94	0.370
B1	−40.32	−122.41, 41.77	0.330
A2	−61.91	−144.00, 20.17	0.137
B2	−103.83	−185.92, −21.75	**0.014**
A3	−77.55	−159.64, 4.53	0.064
B3	−122.87	−204.95, −40.78	**0.004**
**OPG/RANKL ratio**			
A1	0.46	0.20, 0.72	**0.001**
B1	0.43	0.17, 0.68	**0.002**
A2	0.57	0.31, 0.83	**<0.001**
B2	0.37	0.11, 0.63	**0.006**
A3	0.34	0.08, 0.60	**0.011**
B3	0.54	0.28, 0.79	**<0.001**

**Table 3:** Pairwise group per timing mean changes (final–initial) comparisons’ *p*-values derived from linear regression models and adjusted for multiple comparison (Bonferroni).

	*p*-Values		
Mean Change	A1 vs. B1	A2 vs. B2	A3 vs. B3
RANKL	>0.999	>0.999	>0.999
OPG	>0.999	0.507	0.497
MCSF	>0.999	>0.999	>0.999
OPG/RANKL ratio	>0.999	0.802	0.857

**Table 4:** Pairwise timing per group mean changes (final–initial) comparisons’ *p*-values derived from linear regression models and adjusted for multiple comparison (Bonferroni).

	*p*-Values					
Mean Change	A1 vs. A2	A1 vs. A3	A2 vs. A3	B1 vs. B2	B1 vs. B3	B2 vs. B3
RANKL	>0.999	>0.999	>0.999	>0.999	>0.999	>0.999
OPG	>0.999	>0.999	0.634	>0.999	>0.999	0.707
MCSF	>0.999	>0.999	>0.999	>0.999	0.642	>0.999
OPG/RANKL ratio	>0.999	>0.999	0.812	>0.999	>0.999	>0.999

**Table 5:** Descriptive statistics (mean and standard deviation) for each measurement by timing and overall for Groups A and B.

Descriptive Statistics	Mean (SD)				
Experimental Group A	Subgroups—Timing				
	**A1—30 d**	**A2—60 d**	**A3—90 d**	**Overall**	***p*** -Value *
Anterior Ratio BS/TS	69.45 (12.37)	75.52 (6.81)	76.30 (11.35)	73.76 (10.62)	0.342
Posterior Ratio BS/TS	78.31 (13.21)	88.99 (3.33)	89.86 (5.22)	85.72 (9.76)	0.079
Anterior CCT (μm)	252.52 (40.04)	202.96 (74.65)	202.27 (52.45)	219.25 (60.74)	0.186
Middle CCT (μm)	163.12 (68.22)	154.40 (58.46)	153.61 (42.75)	157.05 (55.95)	0.950
Posterior CCT (μm)	97.02 (24.66)	78.77 (30.65)	76.61 (17.56)	84.13 (25.87)	0.516
Weight final (grams)	256.08 (24.46)	320.67 (24.97)	337.00 (58.63)	304.58 (52.15)	<0.001
**Control Group B**	**Subgroups—Timing**				
	**B1—30 d**	**B2—60 d**	**B3—90 d**	**Overall**	***p*** -Value *
Anterior Ratio BS/TS	80.32 (6.81)	83.37 (8.15)	84.20 (7.48)	82.63 (7.48)	0.862
Posterior Ratio BS/TS	90.45 (6.20)	93.51 (6.18)	94.15 (4.60)	92.70 (5.78)	0.576
Anterior CCT (μm)	196.78 (36.54)	166.33 (23.12)	165.78 (17.71)	176.30 (30.03)	0.708
Middle CCT (μm)	177.12 (54.72)	151.71 (29.35)	150.94 (29.06)	159.93 (40.37)	0.660
Posterior CCT (μm)	153.39 (35.55)	143.25 (14.65)	141.63 (14.34)	146.09 (23.60)	0.910
Weight final (grams)	282.25 (18.14)	365.25 (34.98)	430.17 (30.11)	359.22 (67.40)	<0.001

* Derived from linear regression models; pairwise comparisons are provided in Table 6 and Table 7.

**Table 6:** Pairwise group per timing measurements comparisons’ *p*-values derived from linear regression models and adjusted for multiple comparison (Bonferroni).

	*p*-Values *		
Measurements	A1 vs. B1	A2 vs. B2	A3 vs. B3
Anterior Ratio BS/TS	**0.021**	0.196	0.416
Posterior Ratio BS/TS	**0.002**	>0.999	>0.999
Anterior CCT	**0.026**	0.468	>0.999
Middle CCT	>0.999	>0.999	>0.999
Posterior CCT	**<0.001**	**<0.001**	**<0.001**
Weight final	0.202	**0.007**	**<0.001**

* Bold *p*-values indicate statistical significance at 5% level.

**Table 7:** Pairwise timing per group measurements comparisons’ *p*-values derived from linear regression models and adjusted for multiple comparison (Bonferroni).

	*p*-Values *					
Measurements	A1 vs. A2	A1 vs. A3	A2 vs. A3	B1 vs. B2	B1 vs. B3	B2 vs. B3
Anterior Ratio BS/TS	0.788	0.705	>0.999	>0.999	>0.999	>0.999
Posterior Ratio BS/TS	0.141	0.198	>0.999	>0.999	>0.999	>0.999
Anterior CCT	0.321	0.496	>0.999	>0.999	>0.999	>0.999
Middle CCT	>0.999	>0.999	>0.999	>0.999	>0.999	>0.999
Posterior CCT	>0.999	>0.999	>0.999	>0.999	>0.999	>0.999
Weight final	**<0.001**	**<0.001**	0.999	**<0.001**	**<0.001**	**<0.001**

* Bold *p*-values indicate statistical significance at 5% level.

## Data Availability

The data presented in this study are available upon request from the corresponding author.
